# Tuning the Selectivity of Methanol Decomposition to Syngas Exploiting the Surface Stability of Ni_3_Sn_2_ Intermetallic Compounds

**DOI:** 10.1021/acsaem.6c00124

**Published:** 2026-04-21

**Authors:** Silvia Mauri, Maryam Abdolrahimi, Alexios P. Douvalis, Farzane Talaee Shoar, Sara Passuti, Paolo Ronchese, Maria Eugênia Fortes Brollo, Regina Ciancio, Danil W. Boukhvalov, Antonio Politano, Davide Peddis, Piero Torelli

**Affiliations:** † 518735 CNR - Istituto Officina dei Materiali, TASC, Trieste I-34149, Italy; ‡ MAX IV Laboratory, Lund University, Lund SE-221 00, Sweden; § Department of Chemistry and Industrial Chemistry & INSTM RU nM2-Lab, 9302 University of Genova, Genova 16146, Italy; ∥ Institute of Structure of Matter, National Research Council, nM2-Lab, Via Salaria, km 29.300, Monterotondo Scalo 00015, Italy; ⊥ Physics Department, 37796 University of Ioannina, Ioannina 45110, Greece; # 18469 AREA Science Park, Padriciano 99, Trieste I-34149 Italy; ∇ College of Science, Institute of Materials Physics and Chemistry, 74584 Nanjing Forestry University, Nanjing 210037, P. R. China; ○ Institute of Physics and Technology, Satbayev University, Ibragimov str.11, Almaty 050032, Kazakhstan; ◆ Department of Physical and Chemical Sciences, University of L’Aquila, L’Aquila, Via Vetoio 67100, Italy

**Keywords:** Intermetallic, operando, spectroscopy, green, catalysis, methanol, syngas

## Abstract

In this work, we explored the catalytic decomposition of methanol to syngas at 300 °C using intermetallic Ni_3_Sn_2_ nanoparticles (NPs) synthesized via a chemical route. Our study employed a comprehensive approach combining *operando* Ambient Pressure soft X-ray absorption spectroscopy with a suite of *ex situ* techniques—including X-ray diffraction, X-ray photoelectron spectroscopy, electron microscopy, and Mössbauer spectroscopy—and density functional theory (DFT) calculations. Consistent with the behavior observed in Ni_3_Sn_2_ single crystals, we found that the Ni–Sn bonds stabilize the unique electronic structure of the intermetallic Ni active sites, even under strongly oxidizing conditions. Additionally, the nanoparticles exhibit a distinctive morphology characterized by a SnO_*x*_-rich protective shell, which further enhances the stability of the Ni sites. These stabilized sites enable the selective decomposition of CH_3_OH into H_2_ and CO while effectively suppressing coke formation, a major limitation of conventional metallic Ni catalysts, which are currently a benchmark for this reaction. Our findings suggest a promising strategy for the design of scalable, stable, and cost-effective Ni-based catalysts, unlocking the full potential of methanol as a liquid, portable hydrogen carrier.

## Graphical abstract

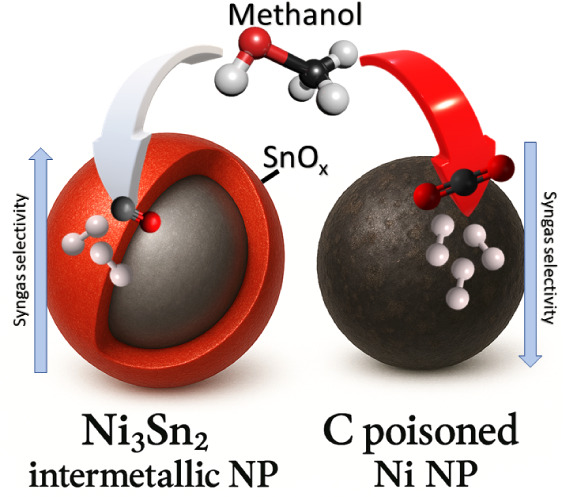

## Introduction

In the green hydrogen economy, methanol is considered one of the most precious chemicals to be exploited in several ways for clean energy production.^1–3^ As a hydrogen-rich compound, liquid at room temperature, methanol is regarded as a valuable solution to the critical issues related to H_2_ transport.^1–3^ Hydrogen production from the direct methanol decomposition reaction (CH_3_OH ⇆ 2H_2_ + CO; see Table S1 for a summary of the state-of-the-art catalysts studied for this reaction) is a promising alternative to the industrially exploited steam reforming of methane (CH_4_ + H_2_O ⇆ 3H_2_ + CO);^1^ its advantage resides in avoiding an external steam and heat source, thereby reducing operating and infrastructure costs. Cheap nickel-based catalysts for the methanol decomposition reaction have been recently investigated, due to the ability of Ni atoms to break C–H and C–O bonds at relatively low temperatures,^4^ making them promising candidates for industrial scale-up.^5–8^ The major drawback of Ni-based catalysts is the occurrence of parallel side reactions such as methanation (CO + 3H_2_ ⇆ CH_4_ + H_2_O), reverse water-gas shift (CO_2_ + H_2_ ⇆ CO + H_2_O), and Boudouard (2CO ⇆ C + CO_2_) reactions, the Boudouard reaction being particularly undesired due to catalyst deactivation caused by coke poisoning.^9,10^ Nickel is well known to strongly adsorb carbon species, initiating the formation of C–C bonds and thereby quickly deactivating the catalyst.^11,12^ For this reason, fine-tuning of Ni electronic properties becomes necessary to design catalysts that combine efficient catalytic activity with affordability and stability. In this context, intermetallic compounds (IMCs)^13–20^ are particularly relevant due to their peculiar electronic structure originating from their atomic arrangement in ordered structures, as opposed to the typical close-packed structure of metallic compounds. Among the IMCs, Ni_*x*_Sn_*y*_ compounds have been reported to suppress secondary reactions in several recent studies regarding methanol decomposition reactions: Sn incorporation indeed can favor hydrogen selectivity and make CO adsorption energetically unfavorable,^21,22^ thus improving catalyst durability. Our recent study^23^ on Ni_3_Sn_2_ and Ni_3_Sn_4_ single crystals revealed that the beneficial effects of Sn incorporation to H_2_ selectivity can be further enhanced by inducing the formation of a SnO_*x*_-rich skin at the intermetallic surface through an oxidation pretreatment at mild temperatures (350 °C). By exploiting these findings and crucially applying them to more scalable catalysts, we synthesized Ni_3_Sn_2_ NPs by a chemical synthetic route,^24^ aiming to obtain an NP design where a core of Ni_3_Sn_2_ intermetallic compound is surrounded by a SnO_*x*_-rich protective layer. The catalytic measurements have shown that Ni_3_Sn_2_ NPs promote methanol decomposition to syngas at 300 °C. Comparing these results with a Ni powder reference, we found that the latter produced a largely over-stoichiometric amount of H_2_ over CO, suggesting strong coke poisoning of the surface, while the Ni_3_Sn_2_ NPs gave a quasi-stoichiometric H_2_/CO ratio, suggesting that no carbon residues were left on the catalyst surface. By exploiting soft X-ray NEXAFS under *operando* conditions, we thoroughly unveiled the electronic structure behavior of the NP chemical species during the catalytic methanol decomposition reaction, directly correlating the catalytic activity and selectivity of the compounds to the spectroscopic evidence. We found excellent electronic structure stability of Sn and Ni atoms, with the latter being actively involved in the interaction with CH_3_OH molecules. By coupling the *operando* NEXAFS results with the catalysis outcomes and theoretical calculations, we confirmed the improved catalyst stability and selectivity due to the suppression of the Boudouard reaction (i.e., coke formation), induced by the peculiar electronic structure of Ni atoms in the Ni_3_Sn_2_ lattice and by the protective and promoter role of the surface SnO_*x*_ layer.

## Results and Discussion

### Ni_3_Sn_2_ Nanoparticle *Ex-Situ* Characterization

Ni_3_Sn_2_ nanoparticles have been synthesized following the preparation method described in detail in the Experimental Methods section. Ni_3_Sn_2_ crystallizes in the orthorhombic *Pnma* [no. 62] space group;^25^ the X-ray diffraction pattern in Figure 1a confirmed the successful synthesis and excluded the presence of other phases in the compound. A morphological analysis conducted with Scanning Electron Microscopy (Figure 1b) evidenced a relatively large size distribution of round-shaped particles, suggesting an aggregation of smaller Ni_3_Sn_2_ crystallites. High-Resolution TEM experiments have been performed for a more precise characterization of the NPs (Figure 1c): the isolated NPs were confirmed to exhibit spherical morphology, as shown in the representative Bright Field image in Figure 1d. We statistically evaluated the NPs’ size distribution, analyzing 850 particles (see Figure S1), and found a median size of 23.73 nm (see Supporting Information for details about the statistical analysis). For a detailed structural characterization of the NPs, 3D Electron Diffraction (3D ED^26^) measurements have been carried out on single, isolated NPs of about 100 nm diameter, using an electron beam size of around 200 nm: the acquired data sets confirm that the NPs are composed of a highly crystalline Ni_3_Sn_2_ single phase matching the structure reported in the literature,^25^ as outlined in the inset of Figure 1d. These observations therefore confirm the nature of the crystal structure composing the NPs and assess the purity of the Ni_3_Sn_2_ phase, excluding the presence of other intermetallic Ni–Sn phases.^27^ Ab initio structure solution was carried out with determination of space group *Pnma* (SG:62), and the resulting structure is highlighted in Figure S2.

**Figure 1:**
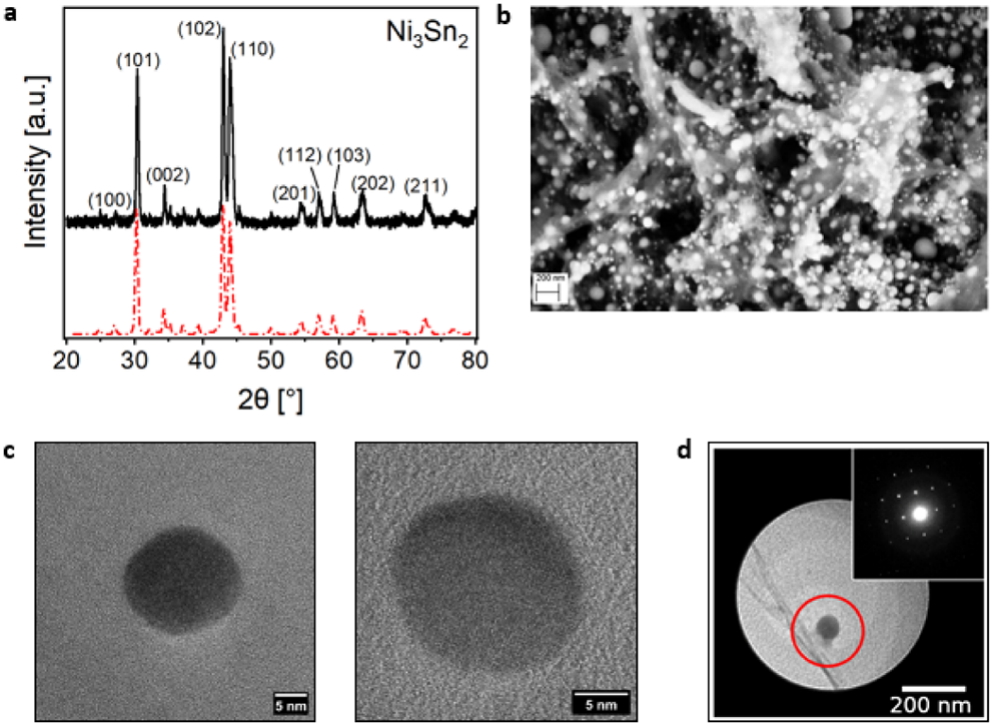
a) XRD pattern of Ni_3_Sn_2_ NPs compared to a reference pattern.^28^ b) SEM image of the Ni_3_Sn_2_ NPs. c) HR-TEM images of two isolated Ni_3_Sn_2_ NPs. d) TEM image of one of the NPs probed by 3D ED, with the red circle representing the approximate size of the used electron beam. In the inset, an example of a diffraction pattern is shown.

The characterization of the pristine Ni_3_Sn_2_ NPs has been complemented by chemical analysis carried out with XPS and NEXAFS analysis in ultra high vacuum (UHV) as well as Mössbauer analysis. The XPS characterization of the NPs (previously exposed to air) in UHV conditions has been conducted to investigate the chemical composition, in particular, of the particles’ surface layers. These measurements have been performed *ex situ* on pristine samples, prior to thermal activation processes and catalytic reactions, and thus are not indicative of the catalyst’s active phases, which have been elucidated extensively by exploiting *operando* NEXAFS spectroscopy, as described in the next section.

The Sn 3d and Ni 2p_3/2_ core-level spectra of Ni_3_Sn_2_ NPs are reported in Figure 2a–b, respectively. From the analysis of the intensities of Sn 3d and Ni 2p core levels based on quantitative XPS, we identified a Sn-rich surface for the NPs; the Sn/Ni ratio in Ni_3_Sn_2_ appears to be 4.46 ± 0.1 (see Supporting Information for details), which is 6.75 times higher than the nominal bulk value of 0.66.

**Figure 2:**
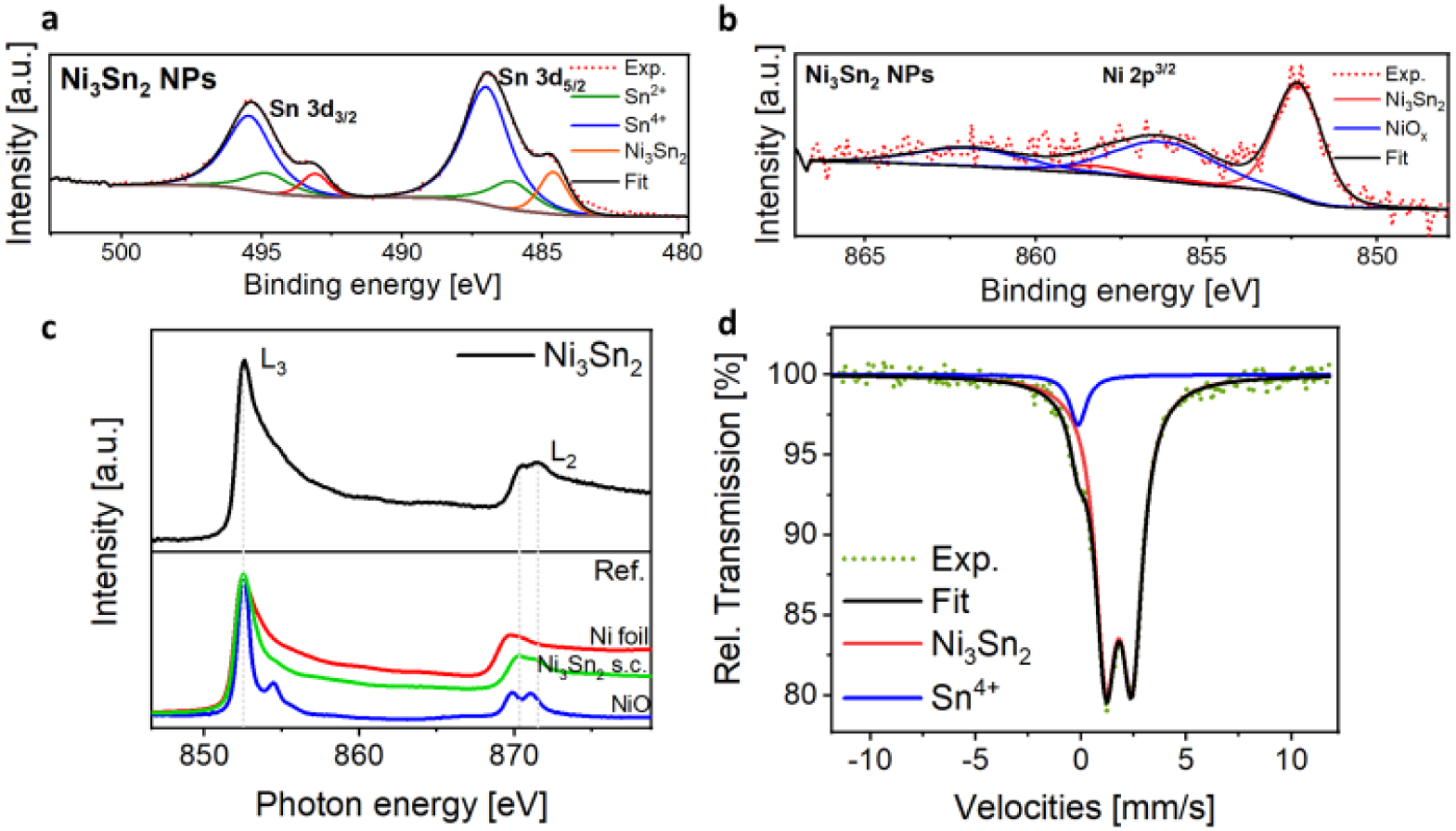
(a–b) X-ray photoemission Sn 3d and Ni 2p core-level spectra of Ni_3_Sn_2_ NPs; c) Top panel: Ni L_2,3_ edges of Ni_3_Sn_2_. Bottom panel: reference spectra of Ni foil, NiO, and Ni_3_Sn_2_ single crystal.^23^ d) Experimental and simulated ^119^Sn Mössbauer spectra of the Ni_3_Sn_2_ NPs at 77 K.

Moreover, curve-fitting analysis of Sn 3d and Ni 2p core-level XPS spectra in Figure 2a–b revealed that Sn atoms are mostly oxidized on the surface of the NPs in the form of Sn^4+^ (70.3%) and Sn^2+^ (16.53%) oxides with only 13% of intermetallic Sn^0^, while Ni atoms maintain a 50% metallic character. In conclusion, the XPS analysis has shown that the surface of the Ni_3_Sn_2_ NPs is Sn-rich (largely above the stoichiometric value) and that Ni and Sn are both partially oxidized due to air exposure, but the Sn-rich skin helps in protecting the metallic Ni from further oxidation. We complemented the chemical *ex-situ* characterization of the NPs with NEXAFS spectroscopy in UHV, which has a higher probing depth compared to XPS (≈5 nm vs ≈1 nm, respectively), in order to investigate the chemical composition of the NPs’ bulk. Figure 2c shows the Ni L_2,3_ edges NEXAFS spectrum of the Ni_3_Sn_2_ nanoparticles compared to the reference spectra of a Ni foil (red line), NiO (blue line), and single-crystal Ni_3_Sn_2_ ^23^ (green line). From the comparison with Ni reference spectra, the white lines clearly show the characteristic features of intermetallic Ni_3_Sn_2_, with no evidence of Ni^2+^ sites. Interestingly, the peculiar electronic structure of this kind of intermetallic compound is more evident in the L_2_ edge, which differs in shape and energy position with respect to Ni^0^ and Ni^2+^, giving a unique fingerprint for Ni_3_Sn_2_. The absence of Ni^2+^ sites once again evidences the role of the SnO_*x*_-rich layer in preventing the oxidation of the inner part of the intermetallic NPs. The Sn M_4,5_ NEXAFS edges reported in Figure S3 appear quite noisy due to the very low absorption cross-section of Sn in its metallic state. Thus, the most visible contribution comes from the Sn^4+^ layer formed at the surface of the NPs as already observed with XPS. Nonetheless, a weak feature at ≈487 eV is still detectable, probably referring to the metallic Sn of the subsurface Ni_3_Sn_2_. To overcome the problem of the low Sn cross-section in NEXAFS and to obtain a bulk characterization of the electronic structure of Sn at this stage, we acquired the ^119^Sn Mössbauer spectrum of the Ni_3_Sn_2_ NPs at 77 K, shown in Figure 2d. The experimental spectrum has been fitted with two simulated components: the main doublet (brown line, 93%) has parameters (IS and QS) that correspond to Sn atoms in the Ni_3_Sn_2_ alloy.^29^ The minor broad component (green line, 7%) has parameters close to those of Sn^4+^ ions in SnO_2_. As with the Sn NEXAFS spectrum, the Mössbauer signal does not perfectly correspond to the SnO_2_ reference due to the peculiar oxide state formed on the NPs’ surface due to air exposure. Combining Mössbauer, NEXAFS, and XPS results, we have been able to clearly define the chemical state of the as-synthesized NPs: they are formed by a thin SnO_*x*_ skin, a poorly crystalline oxide form in which Sn has a + 4 oxidation state, which protects the intermetallic Ni_3_Sn_2_ core from strong Ni oxidation. Given the previous air exposure, surface hydroxides are likely to be present on the surface of the SnO_*x*_ skin of the pristine sample.

### Chemical Reactivity toward Methanol

By exploiting the soft X-ray *operando* NEXAFS technique, we explored the electronic structure evolution of Ni and Sn atoms in Ni_3_Sn_2_ NPs under working conditions for the catalytic CH_3_OH decomposition reaction. Once the sample was mounted in the reaction cell (details are provided in the Supporting Information), the experiment comprised three main steps: i) Acquisition of Ni L_2,3_ and Sn M_4,5_ edges NEXAFS spectra in a He(100%) atmosphere; ii) pretreatment in a He(80%)/O_2_(20%) environment performing a heating ramp from 25 to 300 °C while continuously measuring NEXAFS spectra; iii) after reaching a stationary temperature of 300 °C, the sample was exposed to a constant He(84%)/CH_3_OH­(16%) mixture for approximately 30 min. Finally, CH_3_OH flow was interrupted, and NEXAFS spectra were continuously measured in a helium atmosphere with a decreasing residual concentration of methanol for the subsequent 15 min. The entire experiment was performed at 1 bar of total pressure. During step iii), the reaction products (H_2_, CO, CO_2_, H_2_O, CH_4_) were detected with an online mass spectrometer. The chosen methanol dilution in He corresponds to the saturation vapor pressure of methanol at ambient temperature and pressure. We intentionally did not increase the methanol concentration to minimize mass-transport limitations and reduce the risk of rapid catalyst deactivation, ensuring that the intrinsic behavior of the material could be properly evaluated. Moreover, for *operando* XAS measurements at the Ni L_2,3_ edges, higher methanol concentrations would likely have resulted in a significantly noisier XAS signal after methanol removal, making it difficult to acquire reliable spectra. As a reference, we repeated the entire experiment on a commercial Ni powder, active^8^ for the catalytic CH_3_OH decomposition reaction. More details about the experimental procedure are provided in the Supporting Information.

Figure 3a shows the *operando* NEXAFS spectra of the Ni_3_Sn_2_ NPs during steps ii) and iii) of the experimental procedure described above. After the heating ramp to 300 °C in an oxidizing atmosphere (black spectrum), we do not observe spectral shape changes with respect to the spectrum acquired at 25 °C in UHV in Figure 2c, reflecting the absence of Ni oxidation at this stage; the electronic structure of Ni in Ni_3_Sn_2_ is thus completely preserved after the preoxidation treatment. Looking at the NEXAFS spectrum of the Sn M_4,5_ edges at the same stage (black spectrum of Figure 3c), we observe that it shows the typical features of SnO. This means that during the heating ramp, a Sn^4+^ → Sn^2+^ reduction occurs, probably due to the desorption of hydroxyls and carbonaceous contaminants present on the pristine sample surface before the heating pretreatment. To summarize, after the oxidation pretreatment, the intermetallic character of Ni sites is totally preserved, while Sn surface sites are stabilized in the form of a SnO-like skin. Conversely, Ni^0^ atoms on the surface of the nickel powder reference are oxidized to Ni^2+^ after the heating ramp in He(80%)/O_2_(20%) mixture (Figure 3b, black spectrum), as evidenced by the presence of the pronounced feature at ≈854 eV.^23^ Proceeding to step iii), i.e., exposure to CH_3_OH, extremely different spectroscopic changes were detected between the intermetallic compound and the Ni reference: nickel atoms are reversibly oxidized to Ni^2+^ during methanol exposure in Ni_3_Sn_2_ NPs. Figure 3a indeed shows that immediately after stopping the CH_3_OH flow (i.e., with a residual concentration of CH_3_OH in the reaction cell), the spectral shape appears drastically changed with the presence of a new feature at ≈854 eV, while after 12 min the typical fingerprint of the intermetallic Ni_3_Sn_2_ electronic structure is restored (blue spectrum). Since Ni L edge spectra were continuously acquired during the CH_3_OH removal, it was possible to follow dynamically the gradual restoration of the intermetallic Ni electronic structure as a function of the decreasing f CH_3_OH concentration, as shown in Figure 3e; the Ni^2+^-related feature gradually disappears as a function of elapsed time. This reversible interaction between the catalyst and methanol was not observed in the case of the Ni powder reference: as shown in Figure 3b, after methanol exposure, its spectrum is irreversibly modified with respect to the starting one. The feature at ≈854 eV becomes less pronounced with respect to NiO, and the intensity ratio between the two features constituting the L_2_ edge-related features changes, as well as the L_3_:L_2_ intensity ratio, which decreased from 2.88 to 2.56. This branching ratio value and the double-peaked L edges are compatible with a local chemical environment where Ni atoms are bonded to carbon,^30^ suggesting coke poisoning of the surface. Interestingly, the linear combination fitting analysis (Figure S8) revealed that in both catalysts the active surface under reaction condition is constituted by Ni sites having mostly metallic (or intermetallic) oxidation state and approximately 10% of oxidized Ni^2+^ and Ni^3+^. This is an interesting result considering the strongly different catalysts initial state (99% of intermetallic Ni for Ni_3_Sn_2_ and only 27% of Ni^0^ for the Ni reference): this indicates a strong Ni^2+^to Ni^0^ reduction of the Ni reference as the sample is exposed to CH_3_OH; on the other hand, Ni_3_Sn_2_ is subjected to a slight reversible oxidation. Considering the main possible reaction intermediates involved (shown in Figure 4e) during the methanol decomposition reaction and the secondary reactions (RWGS, Boudouard, methanation), we assigned the Ni^2+^/Ni^3+^ to Ni^0^ reduction observed in the Ni reference to different possible processes: i) H_2_ adsorption, not beneficial since hydrogen is the aimed product; ii) Coke poisoning, strongly not beneficial since it deactivates the catalyst; iii) CO chemisorption, deactivating the catalyst if irreversible (as in the case of the Ni reference); iv) CH_3_ chemisorption, related to methanation secondary undesired reaction. We assigned the Ni^0^/Ni_3_Sn_2_ oxidation to Ni^2+^/Ni^3+^ to: i) Methanol decomposition intermediates (CH_3_O*, CH_2_O, HCO); ii) Adsorption of water, indicating that RWG is occurring; iii) Adsorption of CO_2_, indicator of secondary reactions occurrence.

**Figure 3:**
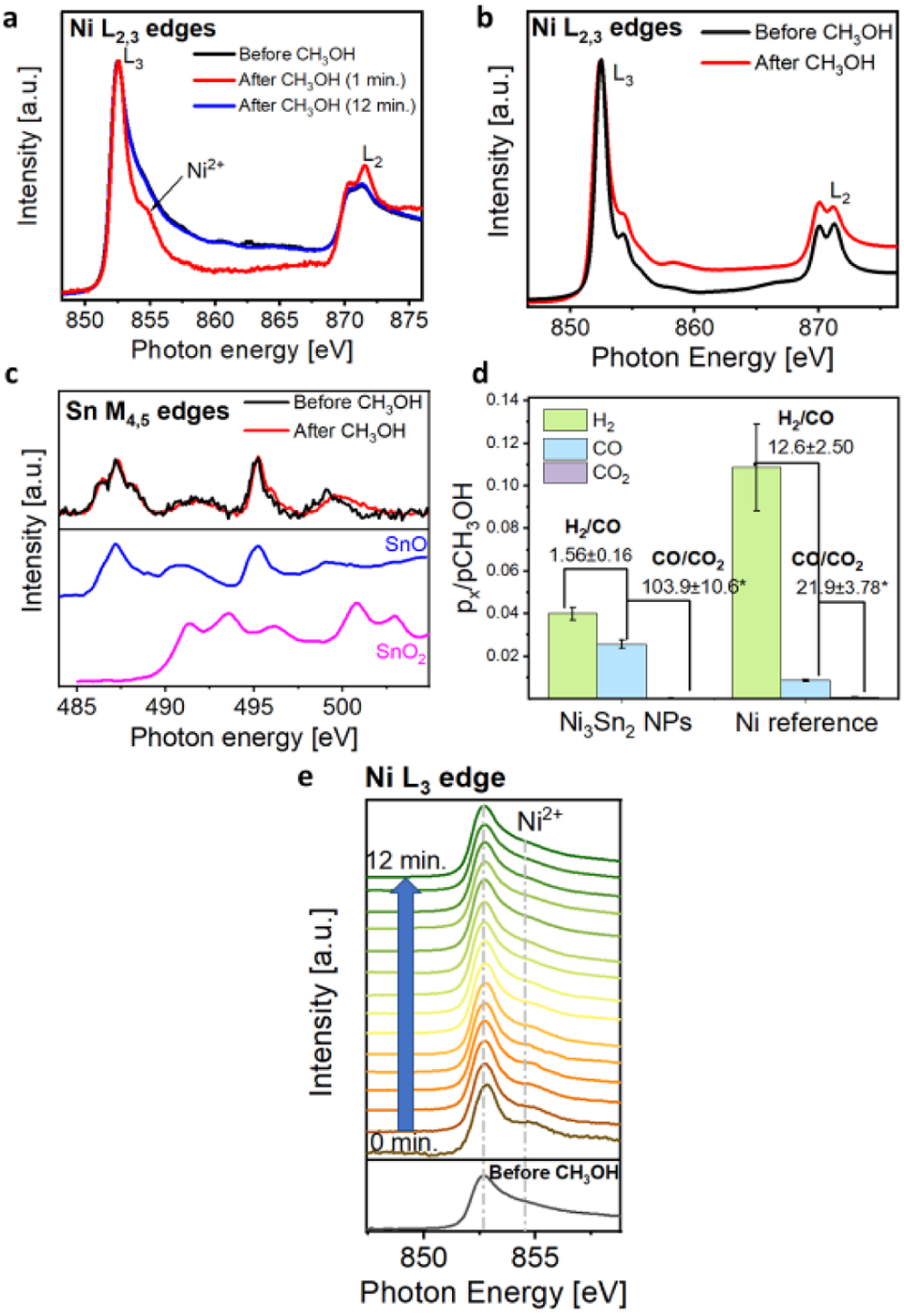
a) Ni L_2,3_ edges spectra of Ni_3_Sn_2_ NPs acquired before exposure to CH_3_OH (black line), 1 min after the removal of CH_3_OH (red line) and 12 min after CH_3_OH removal (blue line). b) Ni L_2,3_ edges spectra of Ni powder reference acquired before exposure to CH_3_OH (black line) and 12 min after CH_3_OH removal (red line). c) Top panel: Sn M_4,5_ edges spectra of Ni_3_Sn_2_ NPs acquired before exposure to CH_3_OH (black line) and 12 min after CH_3_OH removal. (red line). d) Syngas and CO_2_ produced by Ni_3_Sn_2_ NPs and Ni reference powder. e) Ni L_3_ edge spectra evolution during CH_3_OH removal (from the bottom to the top). First spectrum: After 0 min; Last spectrum: After 12 min. Black spectrum: Ni L_2,3_ edges acquired after preoxidation treatment and before CH_3_OH exposure.

**Figure 4:**
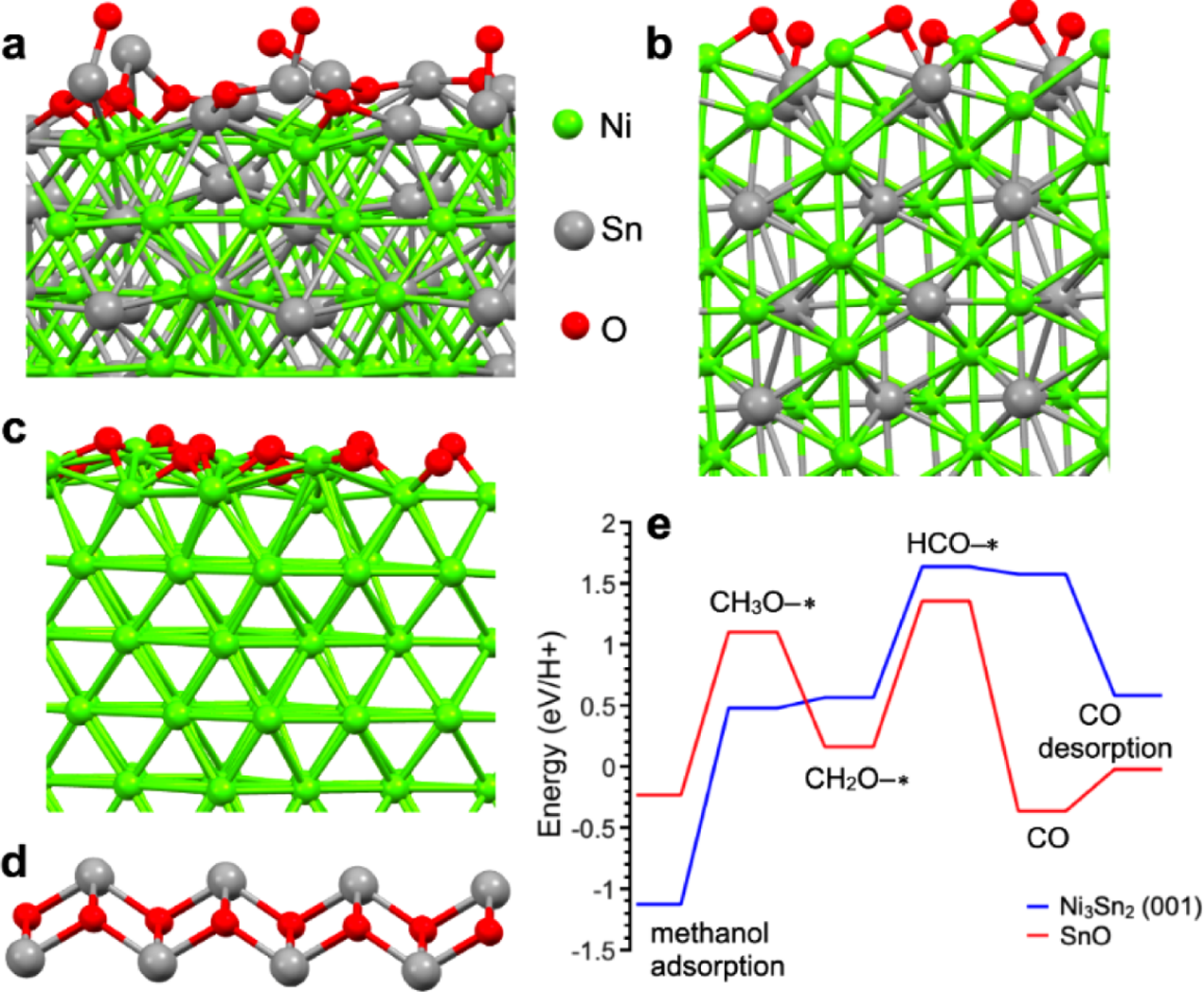
Optimized atomic structure of a) oxidized (001) surface; b) oxidized (010) surface of Ni_3_Sn_2_; c) oxidized (111) surface of Ni; d) surface layer of SnO; e) Free energy diagram for MDR over two stable, carbon-poisoning surfaces. An asterisk marks the substrates.

The comparison of the Sn M_4,5_ edges NEXAFS spectra before and after methanol exposure (Figure 3c) revealed a strong stability in the electronic structure of the SnO skin on the surface of the Ni_3_Sn_2_ NPs upon CH_3_OH interaction.

The analysis of the online mass spectrometer results allowed us to correlate the NEXAFS spectra evolution to the catalytic activity of Ni_3_Sn_2_ NPs and the Ni reference. The main products observed during CH_3_OH exposure at 300 °C for both samples were H_2_ and CO, while secondary products resulting from side reactions (H_2_O, CH_4_, and CO_2_) were detected in lower amounts (see Figure S6). The detection of these products confirmed that for both samples, the catalytic methanol decomposition reaction to syngas successfully occurred. A more detailed semiquantitative analysis of the products by mass spectrometry allowed us to compare the selectivity of Ni_3_Sn_2_ and the Ni reference; we first analyzed the ratio between the produced H_2_ and CO, which was quantified as 1.56 for Ni_3_Sn_2_ and 12.6 for the Ni reference, as reported in Figure 3d. If we consider the mass correction factors of the mass spectrometer analyzer (i.e., ≈2 for H_2_ and ≈1 for CO), H_2_/CO ratio increases to ≈3 for the Ni_3_Sn_2_ NPs and to ≈25 for the Ni reference. In both cases (with or without the correction factors), the values obtained for the Ni_3_Sn_2_ NPs are close to the theoretical stoichiometric value of 2 resulting from the methanol decomposition reaction; this indicates that secondary reactions should be limited on Ni_3_Sn_2_ surface, as also demonstrated by the high ratio between produced CO and CO_2_ which highlights the suppression of the Boudouard reaction. In this semiquantitative evaluation, we did not consider the possibility of the occurrence of the inverse Boudouard reaction (CO_2_ + C ⇌ 2CO), since it is particularly unfavorable under the experimental conditions employed. In the case of the Ni reference, the situation is different: as expected, Ni is active for methanol decomposition as evidenced by the high amount of produced H_2_; at the same time, nickel is known to be easily poisoned by CO and C resulting from the Boudouard reaction.^11,12^ Moreover, the surface sensitivity of NEXAFS allowed us to detect that the surface of the Ni reference powder is oxidized to NiO after the thermal pretreatment in an oxidizing atmosphere. The different electronic structure of the active sites, and thus the different oxidation state, could also contribute to explaining the largely over-stoichiometric H_2_/CO ratio found for the Ni reference: a part of the produced CO is chemisorbed at the Ni surface, while another part is converted to CO_2_ and C. This is further supported by the lower CO/CO_2_ ratio found for the Ni reference (21.9, see Figure 3d). The *operando* NEXAFS spectra acquired consistently reflect the catalytic results just described: for Ni_3_Sn_4_ NPs, Ni atoms reversibly interact with methanol, as evidenced by the total recovery of the electronic structure during CH_3_OH removal, i.e., as a function of the gradual decrease in methanol concentration, as shown in Figure 3a and e. Moreover, Sn M_4,5_ edges NEXAFS spectra also highlighted that the electronic structure of the SnO_*x*_-rich shell of the Ni_3_Sn_2_ NPs is totally preserved after CH_3_OH exposure. SEM analysis after the reaction evidenced that the morphology of the NPs is also preserved after the reaction (see Figure S6). All experimental results point to consistent stability and resistance to carbon poisoning for the SnO_*x*_/Ni_3_Sn_2_ NPs.

In order to further support the obtained results and provide more insights into the different catalytic paths involved during the spectroscopic measurements under *operando* conditions, we modeled, through DFT calculations, the energies involved in the decomposition of CH_3_OH and coke poisoning on different surfaces.

Based on our previous work on similar materials^31^ and XPS, the first step of our modeling was to check the stability of the Ni_3_Sn_2_ surfaces to oxidation. For this purpose, we calculated the free energy of oxygen adsorption from the air at room temperature and the energy of the following decomposition of this molecule (Table 1). The calculations show stable physical adsorption with energy below −20 kJ mol^–1^ for both the (001) and (010) surfaces. The following decomposition is extremely energetically favorable for both surfaces, with an energy gain of more than 80 kJ mol^–1^. The oxidation of the whole surface with the formation of the structures depicted in Figure 4a–b is also a very energetically favorable process for both surfaces, yielding an energy gain exceeding 200 kJ mol^–1^. It is important to underline at this stage that the oxidized (001) surface (model shown in Figure 4a) is Sn–O terminated, while the oxidized (010) surface is both Sn–O and Ni–O terminated: thus, we considered the oxidized (001) surface as the most representative model for the oxidized Ni_3_Sn_2_ NPs. Further increase in the oxygen content leads to the formation of oxide-like structures.

**Table 1:** Energies of the Physical Adsorption of CO from Liquid, Boudouard (B), and Mars-van Krevelen (MvK) Reactions, and the Rate-Determining Step (RDS) of the Methanol Decomposition Reaction (MDR)

Substrate		dE_CO ads_, kJ mol^–1^	dE (B), kJ mol^–1^	dE (MvK), kJ mol^–1^	MDR RDS, kJ mol^–1^
Ni_3_Sn_2_ (001)	Pristine	–55.9	88.1		157.6
	Oxidized	–205.6	–135.1	–116.7	123.9
Ni_3_Sn_2_ (010)	Pristine	–48.7	–107.2		37.2
	Oxidized	–236.3	–333.9	–208.1	44.8
Ni/NiO		–126.6	–811.4	366.4	616.1
SnO		2.2	201.8	45.4	130.0

In addition to pure and oxidized surfaces of Ni_3_Sn_2_, the oxidized (111) surface of nickel and a surface layer of SnO (see Figure 4a–d) have been chosen based on the experimental results to simulate carbon poisoning. For these surfaces, we simulate CO physical adsorption and the Boudouard reaction. For the oxidized surfaces, surface reduction originating from the Mars-van Krevelen reaction (CO + MO ⇆ CO_2_ + MO_1–*x*_) has also been considered as a possible reaction path. Calculated energies for these reactions are summarized in Table 1. These numbers demonstrate that among the six considered substrates, only two are stable to CO-poisoning or CO-induced reduction: the pristine (001) surface of Ni_3_Sn_2_ and SnO. For other surfaces, the Boudouard reaction is exothermic: notably, among the oxidized Ni_3_Sn_2_ surfaces, the reaction is less favored on the (001), i.e., the Sn–O terminated surface. In good agreement with experimental results, the Boudouard reaction is 6 times less favored on the oxidized Ni_3_Sn_2_ (001) surface compared to the Ni/NiO one. Looking at the energetics for MDR, the Ni_3_Sn_2_ (001) surface and SnO show similar results. However, the reaction over SnO is slightly less energetically costly (Figure 4d). Thus, based on the results of the calculations, we propose that a SnO-like layer (hypothetically with a thickness comparable to the oxidized Ni_3_Sn_2_ (001) model of Figure 4a or slightly thicker, approaching the model of Figure 4d) formed on the surface of Ni_3_Sn_2_ is stable and that SnO_*x*_/Ni_3_Sn_2_ NPs are compatible with the selective methanol decomposition to syngas experimentally observed through *operando* NEXAFS. Moreover, DFT results suggest that the SnO_*x*_-like surface layer not only protects the underlying Ni atoms but could act as an active site too, promoting a Boudouard-free reaction path in synergy with intermetallic subsurface Ni active sites. The above discussion is largely supported by the quantitative linear combination fitting analysis shown in Figure S8: the nickel reference gets strongly and irreversibly reduced (≈90%) during and after the exposure to methanol. In the case of Ni_3_Sn_2_, the surface gets slightly oxidized during methanol exposure (≈10%), indicating that CH_3_OH is decomposing on the sample surface. The fitting procedure does not indicate surface poisoning and most importantly, after CH_3_OH exposure the surface electronic structure of the active Ni sites gets recovered, indicating a good restoring of the catalyst initial state.

## Conclusion

In conclusion, this study reports the successful synthesis of Ni_3_Sn_2_ intermetallic nanoparticles that are active for catalytic decomposition reactions at low temperatures (300 °C). Most importantly, through *operando* NEXAFS spectroscopy, we demonstrated that the unique electronic properties of Ni–Sn intermetallic compounds coupled with the specific design of the NPs, i.e., a SnO_*x*_-like surface layer working in synergy with the metallic Ni_3_Sn_2_ subsurface, can play a pivotal role in tuning the selectivity of the reaction toward syngas production. This NP design combines the protective role of the SnO_*x*_ surface skin, which avoids the oxidation of the intermetallic Ni subsurface active sites, with the synergic active role of Sn atoms in effectively suppressing the Boudouard reaction, thereby increasing the catalyst’s stability. The NEXAFS spectra acquisition conducted under *operando* conditions coupled with mass spectrometry allowed us to monitor the electronic structure of the catalyst during its activation and its reaction with methanol. We found that the catalyst surface in both catalysts (Ni_3_Sn_2_ and nickel reference) under the exposure of CH_3_OH shows a mostly metallic oxidation state with ≈10% of oxidized nickel sites related to the adsorption of methanol decomposition intermediates. The experimental results obtained, coupled with DFT modeling, revealed that stable intermetallic Ni active sites interact with methanol molecules without being poisoned by secondary byproducts, while the very stable surface SnO_*x*_ layer serves as a coke poisoning suppressor as well as a promoter. Our findings about the catalytic activity and catalytic reaction mechanism of these intermetallic compounds represent a fundamental basis for the engineering of new low-cost and scalable Ni-based catalysts for hydrogen production via methanol decomposition at low temperatures, with potential applications for the conversion of other H-rich hydrocarbons.

## Experimental Methods

### Ni_3_Sn_2_ Nanoparticles Synthesis

Nickel­(II) acetylacetonate (Ni­(acac)_2_·H_2_O, 95%, Sigma-Aldrich), tin­(II) acetate (Sn­(oac)_2_, 95%, Alfa Aesar), tri-n-octylphosphine (TOP, 97%, Strem), oleylamine (OAm, 90%, Sigma-Aldrich), oleic acid (OAc, Sigma-Aldrich), and borane tert-bu-tylamine complex (TBAB, 97%, Sigma-Aldrich) were used as received without any further purification. Chloroform and acetone were of analytical grade and were purchased from various sources.

For the synthesis of the Ni_3_Sn_2_ NPs, 7 mL OAm, 0.1 mmol Ni­(acac)_2_, 0.1 mmol Sn­(oac)_2_, and 0.15 mL OAc were loaded into a 100 mL three-necked balloon and degassed under vacuum at 80 °C for 2 h while being strongly stirred using a magnetic bar. Afterward, 1 mL of TOP was injected into the solution, while a gentle flow of gas containing Ar/H_2_ 5% was introduced. Subsequently, the reaction flask was heated to 180 °C within 20 min, followed by the quick injection of 1 mmol TBAB in 1 mL degassed OAm solution. At this moment, the color of the reaction immediately changed from light blue to black. Then the reaction was maintained at this temperature for 1 h. At the end of the reaction, it was quenched using a water bath. NiSn NPs were collected by centrifuging and washing the solid product with acetone (as the precipitation solvent) and chloroform (as the dispersion solvent) with a ratio of 9:1. The washing procedure is repeated three times. The as-prepared NPs were finally dried under vacuum at room temperature overnight. Finally, to remove the possible oxide phases, the sample is treated under H_2_ at 800 °C for 30 min.

### Ni Reference Sample

Commercial nickel nanopowder was purchased from Sigma-Aldrich. The average size of the particles is <100 nm.

### X-ray Diffraction

Powder X-ray Diffraction (XRD) patterns were recorded from the as-synthesized NPs using a MiniFlex (Rigaku) diffractometer equipped with a Cu Kα anode (λ = 1.54184 Å) in the 2θ geometry in the range of 20–80 degrees. The size of crystallites (DXRD) in the synthetic product was calculated using the Scherrer formula:

DXRD=0.94λ/Bcosθ

B is the full width at half-maximum (fwhm) estimated after fitting the X-ray diffraction peaks with the pseudo-Voigt function, and θ is the Bragg angle corresponding to the peak position.

### Mössbauer Spectroscopy

The ^119^Sn Mössbauer spectroscopic investigations were carried out with a gamma-ray source of Ca119mSnO_3_. The measurements were conducted with the usual transmission geometry at 77 K.

### X-ray Photoemission Spectroscopy

X-ray photoelectron spectroscopy (XPS) experiments were performed at the laboratories of the Advanced Photoelectric Effect–High Energy (APE-HE) beamline at Elettra Synchrotron in Trieste (Italy) exploiting a conventional non-monochromatized X-ray source (Al Kα = 1486 eV) with a hemispherical electron energy analyzer.^32,33^ The Ni_3_Sn_2_ powders were glued onto the sample holder using conductive silver paste. The samples were positioned at 45° with respect to the incident beam, probing an area of ∼1 mm^2^ and a depth of ∼1 nm. The Ni 3p and Sn 3d core levels were acquired using a pass energy of 50 eV and a dwell time of 1000 ms; they were aligned using the Au VB spectra of a reference Au foil positioned just above the sample. The data analysis (including the energy alignment and fitting process) was performed using CasaXPS^34^ software. The Ni 3p and Sn 3d core levels were fitted using Doniach-Sunjic and Voigt line shapes, respectively.

### *Operando* XAS

The measurements were performed at the APE-HE beamline of the Elettra Synchrotron in Trieste (Italy). The beamline is equipped with a proper setup designed to acquire XAS spectra continuously at 1 bar, as depicted in Figure S4 of the Supporting Information. The reaction cell, described in detail elsewhere,^35^ is directly connected to a mass spectrometer. This allows performing *operando* measurements since it is possible to monitor the gaseous products obtained from the catalytic reactions occurring inside the reaction cell. The spectra were acquired in Total Electron Yield (TEY) mode. Once the samples were loaded into the reaction cell, the reactor was filled with helium. The gases were injected into the cell through three flowmeters, calibrated to a maximum total flow of 50 mL/min. In order to inject the methanol, a glass bubbler was added to the gas line, using He as an inert carrier gas. The experimental procedure followed for the *operando* XAS experiments is reported in detail in the Supporting Information.

### Mass Spectrometry

The *operando* XAS reaction cell was equipped with a Pfeiffer Omnistar Mass Spectrometer in order to semiquantitatively detect the reaction products and reactants. The instrument was equipped with a quadrupole mass analyzer that ionizes and separates various molecules based on their different *m*/*z* values. In order to compare the different activity and selectivity of Ni_3_Sn_2_ and reference Ni powder, the signals of the produced H_2_, CO, and CO_2_ were normalized by the reactant (CH_3_OH) signal and by the catalyst weight. Since the instrument was not previously calibrated, the mass correction factors provided by Pfeiffer Vacuum were applied to the obtained values.

### HR-TEM

A small amount of Ni_3_Sn_2_ NPs in powder form was collected and transferred into a 2 mL plastic vial, to which approximately 1 mL of CHCl_3_ was added. The resulting mixture was sonicated for 15 min at room temperature. The suspension was therefore drop-cast on a 3 mm carbon-coated copper grid. High-Resolution TEM and Electron Diffraction experiments have been carried out by using a JEOL JEM-F200 Cold FEG Transmission Electron Microscope operated at 200 kV. HR-TEM images have been recorded through a TVIPS TemCam-XF416 camera equipped with a 4k × 4k CMOS sensor and controlled by EM-Menu 5 software. Single-tilt diffraction patterns have been acquired with a step of 0.5° in an angular range of 50° and recorded with a Dectris ELA hybrid pixelated detector controlled by the CEOS Panta Rhei software.

HR-TEM images were analyzed through ImageJ software. ED data sets have been processed using PETS2.0 ^36^ software for the determination of the unit cell and integration of the diffracted intensities, while Jana2020 ^37^ has been employed for ab initio structure solution using the Charge Flipping algorithm.^38^

### Theoretical Model

The atomic structure and energetics of various configurations were studied by DFT using the QUANTUM-ESPRESSO code^39^ and the GGA-PBE,^40^ taking into account van der Waals forces correction.^41^ For all calculations, ultrasoft pseudopotentials^42^ were used. The values of energy cutoffs were 35 and 400 Ry for the plane-wave expansion of the wave functions and the charge density, respectively. The enthalpy of the reaction is defined as the difference in the calculated total energies of the products and the reactant. Thus, a negative enthalpy indicates exothermic reactions.

The standard formula calculated physisorption enthalpies:

1 $$\Delta H_{phys}=\left[E_{host+mol}-\left(E_{host}+E_{mol}\right)\right]$$

where E_host_ is the total energy of a pristine surface, and E_mol_ is the energy of the single molecule of the selected species in the empty box. In the case of water adsorption, only the gaseous phase was considered. For the case of physisorption, we also evaluated the differential Gibbs free energy by the formula:

2 ΔG=ΔH−TΔS

where T is the temperature, and ΔS is the change in entropy of the adsorbed molecule, which was estimated considering the gas → liquid transition by the standard formula:

3 $$\Delta S=\Delta H_{vaporization}/T$$

where ΔH_vaporization_ is the measured enthalpy of vaporization.

## Supplementary Material



## Notes

Additional experimental details, data analysis explanations, and supplementary experimental results, including schemes of the experimental setup (PDF)
