# Supplementary material for: Tuning the Selectivity of Methanol Decomposition to Syngas Exploiting the Surface Stability of Ni3Sn2 Intermetallic Compounds
Source: ACS Appl Energy Mater. 2026 Apr 21;9(9):5676–84. doi: 10.1021/acsaem.6c00124 (PMC13169341; doi:10.1021/acsaem.6c00124)
Supplement: Supplementary file 1 [file ae6c00124_si_001.pdf]

Supporting information

Tuning the selectivity of methanol decomposition exploiting the surface stability of Ni_3Sn_2 intermetallic compounds

Silvia Mauri[a,b] , Maryam Abdolrahimi[c,d], Alexios P. Douvalis[e], Farzane Talaee Shoar[c,d], Sara Passuti[f], Paolo Ronchese[a,f], Maria Eugênia Fortes Brollo[a,f], Regina Ciancio[a,f], Danil W. Boukhvalov[g,h], Antonio Politano[i], Davide Peddis[c,d], Piero Torelli[a*]*

[a] CNR - Istituto Officina dei Materiali, TASC, I-34149 Trieste, Italy.

[b] MAX IV Laboratory, Lund University, SE-221 00 Lund, Sweden.

[c] Department of Chemistry and Industrial Chemistry & INSTM RU nM2-Lab, University of Genova, 16146, Genova, Italy.

[d] Institute of Structure of Matter, National Research Council, nM2-Lab, Via Salaria, km 29.300, Monterotondo Scalo, 00015.

[e] Physics Department, University of Ioannina, 45110 Ioannina, Greece.

[f]AREA Science Park, Padriciano 99, Trieste, I-34149 Italy.

[g] College of Science, Institute of Materials Physics and Chemistry, Nanjing Forestry University, Nanjing 210037, P. R. China

[h] Institute of Physics and Technology, Satbayev University, Ibragimov str.11, Almaty 050032, Kazakhstan.

[i] Department of Physical and Chemical Sciences, University of L'Aquila, Via Vetoio, 67100 L'Aquila, Italy.

Corresponding Authors

*Silvia Mauri (mauri@iom.cnr.it), *Piero Torelli (piero.torelli@cnr.it)

Table 1- Summary of the state- of-the-art catalysts studied for methanol decomposition catalytic reaction.

**Catalyst**	**Reaction Temperature**	**Data on performance**	**Comments**	**Reference**
Pd/CeO_2	200°C	H_2 and CO selectivity = 99%	Noble metal, carbon coking	[1]
Pt/CeO_2	300°C	TOF 84,225 h^{-1}	Noble metal	[2]
Pt and Pd supported catalysts	220°C	Max CH_3OH = 50%	Noble metals	[3]
Pt/$Ce_{0.75}Zr_{0.25}O_2$ and Pt/$Ce_{0.75}Zr_{0.25}O_2$ –Al_2O_3/FeCrAl	400°C	CH_3OH conversion 100%	Noble metals	[4]
Ni supported NPs	350°C	Selectivity 80% at 100% CH_3OH conversion	No info about stability and coke deposition.	[5]
Ni supported hydrotalcite	450°C	H2 max yield= 73%	Coke deposition	[6]
Ni_3Al	250°C-360°C	At 350°C, H_2 production rate = 350 cm3/min*g_{cat}		[7]
SnO	375°C	CH_3OH conversion = 15%	Not selective for H_2. Selective to CO_2, CH_4 and CH_2O	[8], [9]
SnO films	300°C	Selectivity to CH_2O, not for H_2.	TPD controlled study on SnO surfaces	[10]
SnO	300°C	100% of methanol conversion in presence of O_2 (methanol oxidation). Low selectivity to H_2 (below 50%)	Methanol oxidation reaction, presence of O2 in the reaction feed	[11]

TEM Characterization

The NPs size distribution has been evaluated on a sample of 850 particles. The histogram shown in Fig. S1 has been fitted with a lognormal function reported hereafter:

$$y = y_0 + \frac{A}{\sqrt{2\pi}wx} \exp\left(-\frac{ln\left(\frac{x}{xc}\right)^2}{2w^2}\right) \text{ (eq.1)}$$

In this function, *xc* is the scale parameter of the lognormal distribution (median value), while *w* is the Shape Parameter, or Logarithmic Standard Deviation.

The lognormal fitting gave values of xc= 23.73 and w= 0.43. The arithmetical mean size of the particles has been also calculated, resulting in an average diameter of 28.3 nm.

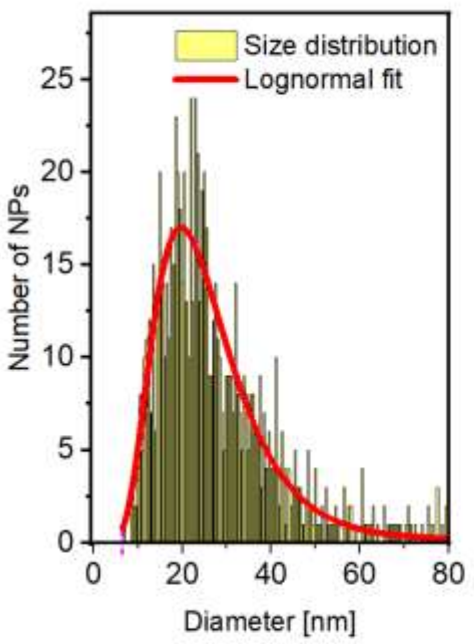

Figure S1. Histogram displaying the size distribution of the batch of 850 NPs analyzed by HR-TEM.

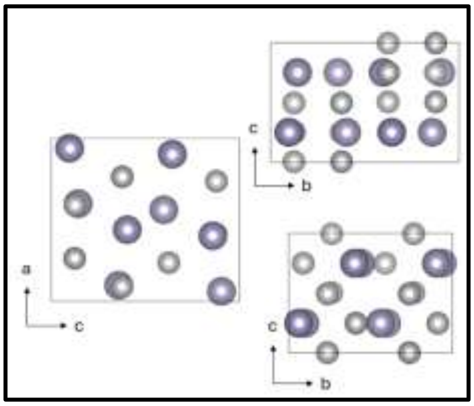

Figure S2. Crystal structure of the Ni_3Sn_2 compound solved ab initio from electron diffraction data on Jana2020, view along [100], [010] and [001] lattice directions. Refined lattice parameters (Å): a=6.84, b=5.30, c=7.75.

Sn/Ni ratio quantitative XPS calculation

A consideration about the stoichiometric composition of the sample can be made, looking at the different intensities of the Ni 2p and Sn 3d core levels of Figure 2 in the main text. The areas of these peaks have been divided for the cross sections (0.34 for the Sn 3d and 0.29 for the Ni 2p, considering a photon energy of 1486 eV [12, 13]) in order to calculate the relative concentration of the two elements, finding out that the Sn/Ni ratio is equal to 4.46 ± 0.1. Given the surface sensitivity of the XPS technique (which at 1486 eV of photon energy should be about few atomic layers), this means that the analyzed sample surface stoichiometry is very different from the Sn/Ni ratio of 0.66 of the bulk.

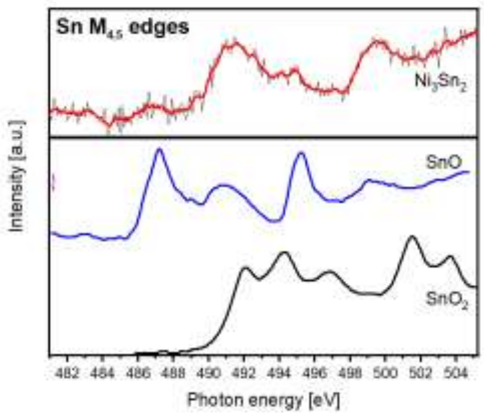

Figure S3: Spectra of Sn $M_{4,5}$ edges: Ni_2Sn_3 NPs black curve experimental, red curve smoothing 12 point (top panel), blue curve SnO reference and black thick curve SnO_2 reference.

The NEXAFS spectra of Sn $M_{4,5}$ edges of the pristine Ni_2Sn_3 NPs acquired in UHV conditions together with the Sn $M_{4,5}$ edges of SnO and SnO_2 reference powders are reported in Figure S3. The spectrum is quite noisy due to the low cross section of Sn, particularly in the metallic state. Despite the noise the spectrum compares quite well with the one of the SnO_2 reference indicating a dominant Sn^{+4} oxidation state. A weak feature at ≈ 487 eV suggests that also a reduced Sn oxidation state, probably the metallic Sn of Ni_3Sn_2, is visible in the NEXAFS spectrum. The small difference between the spectra of the NPs and the SnO_2 reference spectra are attributed to the fact that the oxide spontaneously formed at the NPs surface has a poor crystallinity and is probably also contaminated by the presence of hydroxides species. Thus, the NEXAFS technique which is highly sensitive to the details of the electronic structure reflects the difference between this SnO_x state and a pure crystalline SnO_2.

Experimental procedure for the *Operando* XAS experiments

Ni_3Sn_2 NPs and Ni reference powder were subjected to the same experimental procedure. Initially, the samples were loaded in the chemical reactor depicted in Figure S4a. The sample mounting has been performed in air, thus the samples have been subjected to air exposure. Once

the *operando* XAS reaction cell has been loaded in the dedicated measurement UHV chamber, the reactor has been filled with He(100%) flow exploiting a dedicated gas line system (Figure S4c) provided with mass flow controllers. XAS measurements are performed exploiting a Si_3N_4 membrane placed between the sample and the UHV environment outside the reaction cell (orange circle in Figure S4a), allowing the x rays to hit the sample maintaining the differential pressure between the reactor (1bar) and the measurement chamber ($1x10^{-5}$ mbar). TEY signal is acquired from an electrical connection placed on the membrane. Then, the materials were heated from room temperature to 300 °C in a He(80%)/O_2(20%) flow mixture, exploiting the external gas line shown in Figure S4. Then, the oxygen was removed from the reaction mixture, until a steady state was reached. Successively, the connection to the bubbler was opened, allowing the methanol vapors to enter the reaction cell for approximatively 30 minutes. Then, the methanol was removed, keeping the samples in He until a steady state was reached. During this stage, NEXAFS spectra of Ni $L_{2,3}$ edges and Sn $M_{4,5}$ edges have been continuously acquired in order to detect reversible modifications induced by methanol presence. For sake of clarity, a graphical summary of the experimental procedure is reported in Figure S5.

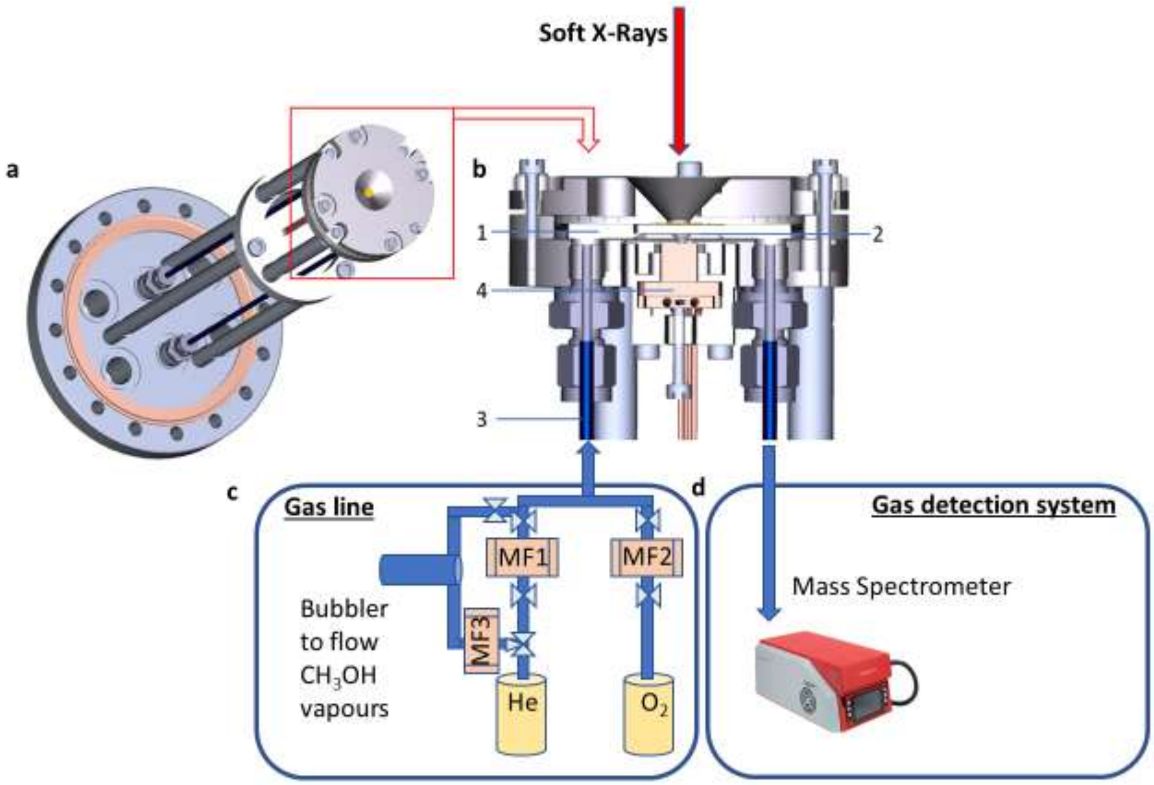

Figure S4 - Custom-made operando soft-NEXAFS setup at APE-HE beamline. a) 3D rendering of the reaction cell; b) Enlarged side view of the reactor: 1- Reactor volume; 2- Sample holder; 3- Gas inlet; 4- Heating system. Directions of incoming X-Rays with respect to the reactor are indicated with a red arrow. c) Gas line provided with two inlets dedicated to He and O_2 gases, with the addition of a bypass line to flow a mixture of He and CH_3OH. D) Gas detection system consisting of a mass spectrometer.

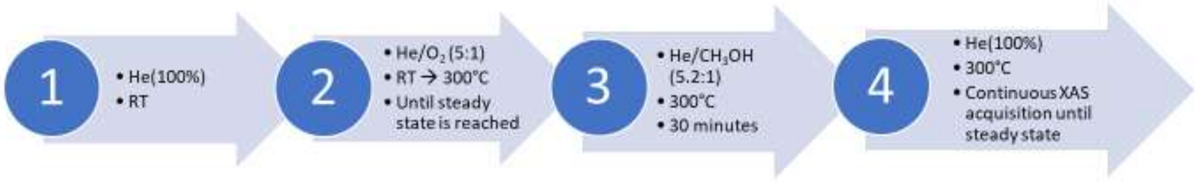

Figure S5 -Experimental procedure followed for the operando NEXAFS experiment conducted on Ni_3Sn_2 NPs and Nickel powder reference.

Mass Spectrometer results

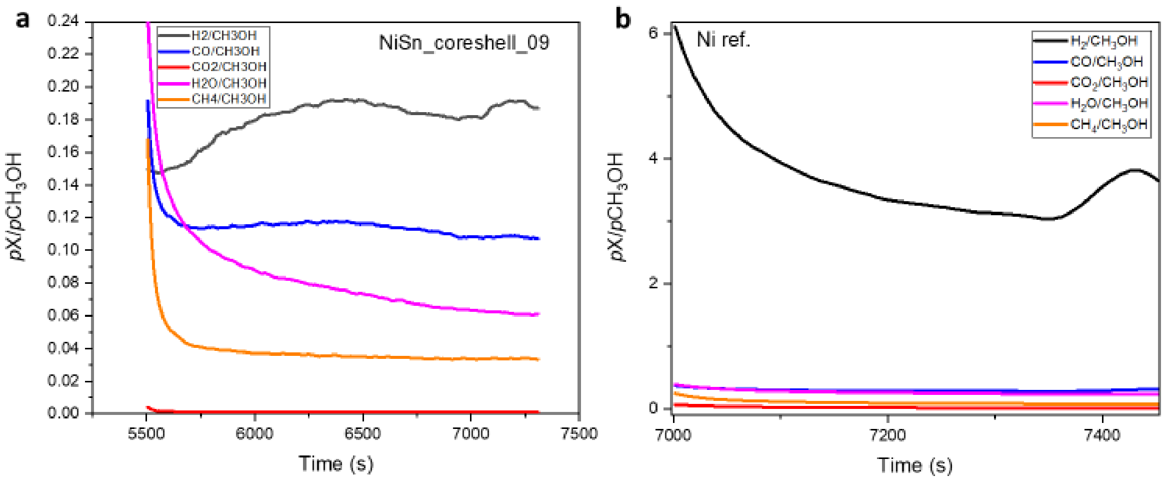

Figure S6 – Mass Spectrometry results obtained for a) Ni_3Sn_2 NPs and b) Nickel reference. These data have not been normalized to the catalysts weight.

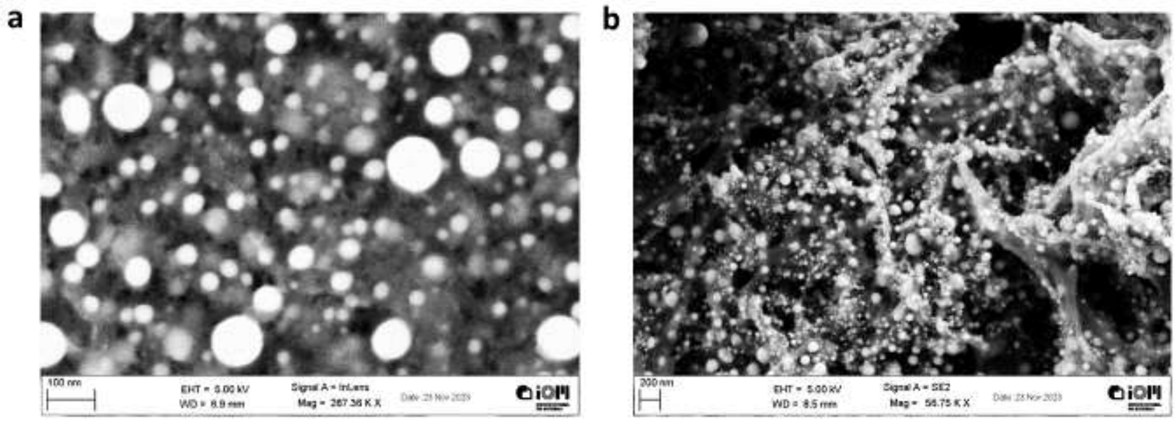

Figure S7 – a-b) SEM images of Ni_3Sn_2 NPs after the operando NEXAFS experiment.

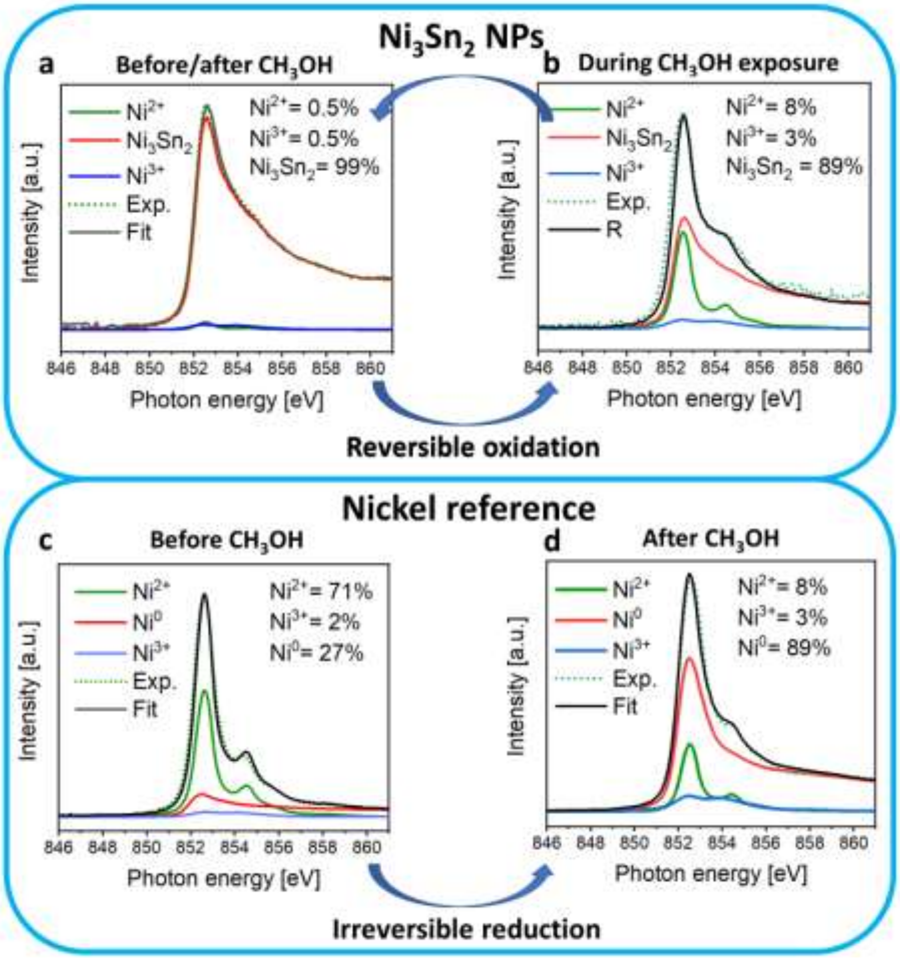

Figure S8 – Linear combination fitting procedure performed on Ni_3Sn_2 spectra (top) and Ni reference spectra (bottom), in different experimental conditions.

Linear combination fitting procedure for *operando* NEXAFS spectra

We performed a linear combination fitting analysis of well defined reference nickel spectra as basis functions, already used successfully in our previous studies [14], according to the following formula:

Fitted spectrum = $a*Ni^{2+} + b*Ni^{3+} + c*Ni^{0/intermetallic}$ (eq. 1)

Thus, the concentration of the different oxidation states of Ni can be obtained as follows:

$\%Ni^{2+} = a/(a+b+c)$ (eq. 2)

$\%Ni^{3+} = b/(a+b+c)$ (eq. 3)

$\%Ni^{0/intermetallic} = c/(a+b+c)$ (eq. 4)

where *a*, *b*, and *c* are coefficients ranging from 0 to 1 depending on the different Ni^x contents.

In order to perform a quantitative evaluation, the area of the reference spectra of Ni^{2+}, Ni^{3+},Ni^0 and Ni_3Sn_2 was chosen in order to take into account their different cross sections. In first approximation the intensity of the white lines of the x ray absorption spectra are proportional to the available state in the conduction band, thus for the L edges of the transition metals which are dominated by the 2p --> 3d transition the intensity is proportional to the number of holes in the 3d shell [15]. Unfortunately, the 3d occupancy is not known with precision and one has to rely on calculation accepting a certain degree of uncertainty. In the present case the needed numbers are the $3d^n$ of Ni^{3+}, Ni^{2+} Ni^0 (metallic Ni) and Ni_3Sn_2. In literature is quite well accepted that Ni^{3+} and Ni^{2+} correspond to $3d^7$ and $3d^8$ respectively. The calculation for metallic Ni indicates $3d^9$ [16]. For Ni_3Sn_2 no numbers are available to our knowledge but we found a recent calculation for Ni_3Sn_4 which indicates an occupation of the 3d of 9.5 [17]: we have used this value as the best approximation for Ni_3Sn_2 3d occupation number.

References

[1] - Carraro, F.; Fapohunda, A.; Paganini, M.C.; Agnoli, S. Morphology and Size Effect of Ceria Nanostructures on the Catalytic Performances of Pd/CeO_2 Catalysts for Methanol Decomposition to Syngas. *ACS Appl. Nano Mater.* **2018** 1 (4), 1492-150. DOI: 10.1021/acsanm.7b00408.

[2] - Liu, Y; Zou, Y; Wang, Y; Ma, Y; Sai Zhang, Qu, Y.; Strong metal-support interactions between Pt and CeO_2 for efficient methanol decomposition, *Chem. Eng. J.* **2023**, 475, 146219, https://doi.org/10.1016/j.cej.2023.146219.

[3] - Iwasa, N.; Takezawa, N. New Supported Pd and Pt Alloy Catalysts for Steam Reforming and Dehydrogenation of Methanol. *Top. Catal.* **2003** 22, 215–224. https://doi.org/10.1023/A:1023571819211.

[4] - Badmaev, S.; Belyaev, V.; Potemkin, D.; Snytnikov, P.; Sobyanin, V.; Kharton, V. Methanol Decomposition to Synthesis Gas over Supported Platinum-Containing Catalysts. *Catal. Ind.* **2023** 15. 367-373. 10.1134/S2070050423040037.

[5] - Tsoncheva, T.; Rosmini, C.; Mihaylov, M.; Henych, J.; Chakarova, K.; Velinov, N.; Kovacheva, D.; Němečková, Z.; Kormunda, M.; Ivanova, R.; Spassova, I.; Hadjiivanov, K. *ACS Appl. Mater. Interfaces* **2022** **14** (1), 873-890. DOI: 10.1021/acsami.1c19584.

[6] - Rojas, H.A.; López, V.P.; Brijaldo, M.H.; Mancipe, S.; Martínez, J.J.; Cortés, A. G.; Araiza, D. G; Díaz, G. Effect of boron on the surface properties of nickel supported on hydrotalcite-type mixed oxides in methanol decomposition, *Mol. Catal.* **2020**, 498, 111262, https://doi.org/10.1016/j.mcat.2020.111262.

[7] - Ma, Y.; Xu, Y.; Demura, M.; Chun, D.H.; Xie, G.; Hirano, T. Catalytic activity of atomized Ni_3Al powder for hydrogen generation by methane steam reforming. *Catal. Lett.* **2006**, 112, 31–36. https://doi.org/10.1007/s10562-006-0160-5.

[8] - Mori, T; Hoshino, S; Neramittagapong, A; Kubo, J; Morikawa, Y. Novel activity of SnO_2 for methanol conversion: Formation of methane, carbon dioxide, and hydrogen. *Chem. Lett.* **2022**, 3, 390-391. 10.1246/cl.2002.390.

[9] - Neramittagapong, A.; Grisdanurak, N.; Neramittagapong, S.; Suppression of CO on methanol decomposition over SnO_2 catalysts, *J. Ind. Eng. Chem* **2008**, 14, 4, 429-435, https://doi.org/10.1016/j.jiec.2008.02.005.

[10] - Gercher, V.A.; Cox, D.F; Themlin, J.M.; Oxygen-vacancy-controlled chemistry on a metal oxide surface: methanol dissociation and oxidation on SnO_2(110), *Surf. Sci.* **1994,** 306, 3, 279-293, https://doi.org/10.1016/0039-6028(94)90072-8.

[11] - Carreño, N.L.V.; Maciel, A.P.; Leite, E.R.; Lisboa-Filho, L. N.; Longo, E.; Valentini, A.; Probst, L.F.D; Paiva-Santos, C.O.; Schreiner, W.H. The influence of cation segregation on the methanol decomposition on nanostructured SnO_2, *Sensors and Actuators B: Chemical* **2002** 86, 2–3, 185-192, https://doi.org/10.1016/S0925-4005(02)00169-7.

[12] - Yeh. J.J.; Atomic Calculation of Photoionization Cross-Sections and Asymmetry Parameters. Gordon and Breach Science Publishers, Langhorne, PE (USA)**, 1993**.

[13] – Yeh J.J.; Lindau I. Atomic Subshell Photoionization Cross Sections and Asymmetry Parameters: $1 \leq Z \leq 103$. *At. Data Nucl. Data Tables* **1985**, *32*, 1-155.

[14] – Felli, A. ; Mauri, S.; Marelli, M.; Torelli, P.; Trovarelli, A.; Boaro, M. Insights into the Redox Behavior of $Pr_{0.5}Ba_{0.5}MnO_{3-\delta}$-Derived Perovskites for CO_2 Valorization Technologies *ACS Appl. Energy Mater.* **2022** 5 (6), 6687-6699. DOI: 10.1021/acsaem.2c00163.

[15] – Graetz, J.; Ahn, C. C.; Ouyang, H.; Rez, P.; Fultz, B. White lines and d-band occupancy for the 3d transition-metal oxides and lithium transition-metal oxides. *Phys. Rev. B* **2004** 69, 23, 235103. 10.1103/PhysRevB.69.235103.

[16] - Pearson, D. H.; Ahn, C. C., Fultz, B. White lines and d-electron occupancies for the 3d and 4d transition metals, *Phys. Rev. B* **1993**, 47, 14, 8471-8478, 10.1103/PhysRevB.47.8471.

[17] - Wang, L.; Huang, Y.; Guo, S.; Yao, J.; Xing, J.; Ma, H.; Wang, Y.; and Chen, J. Effect of Different Concentrations of Co Doping on the Properties of η'-Cu_6Sn_5 and Ni_3Sn_4: First-Principles Study. *Phys. Status Solidi B* **2024**, 261: 2400106. https://doi.org/10.1002/pssb.202400106.
